# Analysis of the relationship between the HbA1c screening results and the development and worsening of diabetes among adults aged over 40 years: a 4-year follow-up study of 140,000 people in Japan – the Shizuoka study

**DOI:** 10.1186/s12889-021-11933-z

**Published:** 2021-10-18

**Authors:** Shuhei Nomura, Haruka Sakamoto, Santosh Kumar Rauniyar, Koki Shimada, Hiroyuki Yamamoto, Shun Kohsaka, Nao Ichihara, Hiraku Kumamaru, Hiroaki Miyata

**Affiliations:** 1 Research Support Center, Shizuoka General Hospital, Shizuoka, Japan; 2 Department of Health Policy and Management, School of Medicine, Keio University, 35 Shinanomachi, Shinjuku-ku, Tokyo, 160-8582 Japan; 3 Department of Global Health Policy, Graduate School of Medicine, The University of Tokyo, Tokyo, Japan; 4 Graduate School of Public Health, Shizuoka Graduate University of Public Health, Shizuoka, Japan; 5 Department of Healthcare Quality Assessment, Graduate School of Medicine, The University of Tokyo, Tokyo, Japan; 6 Department of Cardiology, School of Medicine, Keio University, Tokyo, Japan

**Keywords:** Japan, Diabetes, Health check-ups, Follow-ups, Claims data

## Abstract

**Background:**

Hemoglobin A1c (HbA1c) levels are routinely measured during health check-ups and are used as an indicator of glycemic control in Japan. However, only a few studies have followed up individuals to assess the risk of diabetes development and worsening based on HbA1c screening results. This study evaluated the relationship between HbA1c screening results and the risk of diabetes development and worsening.

**Methods:**

Data were collected from the Shizuoka Kokuho Database, a Japanese administrative claims database of insured individuals aged > 40 years. We included individuals available for follow-up from April 2012 to March 2018 who had not received any diabetes treatment before March 2014. HbA1c screening results were categorized into 4 groups based on the HbA1c levels at the 2012 and 2013 health check-ups: group A, those whose HbA1c levels were < 6.5% in 2012 and 2013; group B, those whose HbA1c levels > 6.5% in 2012 but < 6.5% in 2013; group C, those whose HbA1c levels were > 6.5% in 2012 and 2013; and group D, those whose HbA1c levels were < 6.5% in 2012 and > 6.5% in 2013. Logistic regression models were used to analyze diabetes development and worsening, defined as the initiation of diabetes treatment by March 2018 and the use of injection drugs by participants who initiated diabetes treatment by March 2018.

**Results:**

Overall, 137,852 individuals were analyzed. After adjusting for covariates, compared with group A, group B was more likely to initiate treatment within 4 years (odds ratio: 22.64; 95% confidence interval: 14.66–34.99). In patients who initiated diabetes treatment by March 2018, injection drugs were less likely used by group D than by group A (odds ratio: 0.28; 95% confidence interval: 0.12–0.61).

**Conclusions:**

Our study suggests that although HbA1c levels measured during health check-ups were correlated with the risk of diabetes development and worsening, HbA1c levels in a single year may not necessarily provide sufficient information to consider these future risks.

**Supplementary Information:**

The online version contains supplementary material available at 10.1186/s12889-021-11933-z.

## Background

According to the latest 2019 Global Burden of Disease (GBD) study, diabetes mellitus is one of the leading causes of disease burden globally [1], with a prevalence of 9.0% in women and 9.6% in men in 2019 [2]. In Japan, the 2018 National Health and Nutrition Survey estimated that the proportion of people strongly suspected of having diabetes was 9.3 and 18.7% in the female and male population, respectively. In addition, with respect to age group, these proportions were higher for older people [3]. The healthcare cost for diabetes in Japan was approximately US$12 billion in 2018, of which US$8 billion was incurred by patients aged ≥65 years. This amount is expected to increase by US$70 million annually for this age group [4].

Diabetes is a major risk factor for various diseases, such as acute myocardial infarction and chronic kidney disease. Healthy lifestyle and early preventive interventions in patients with pre-diabetes to prevent the development or worse prognosis of diabetes appear to be one of the most cost-effective measures as severe cases of diabetes increase the development of these diseases and incur higher healthcare costs [5, 6]. Various attempts have been made to estimate the risk of developing diabetes. For example, a study on Japanese population reported that hypertension, fatty liver, body mass index (BMI), and weight-gain percentage since 20 years old are predictors of diabetes incidence [7–9]. In 2018, Japan’s National Center for Global Health and Medicine developed models for predicting the onset of diabetes within 3 years, which was based on medication history for hypertension and hyperlipidemia and BMI [10]; however, the predictive accuracy of these models has not yet been fully evaluated.

Health check-ups are widely available to the general Japanese population aged ≥40 years and play a vital role in screening for diabetes and other lifestyle-related diseases. Fasting blood glucose and hemoglobin A1c (HbA1c) levels are routinely measured during health check-ups to screen for diabetes; however, only a few studies have followed up individuals to assess their risk of diabetes development of worsening based on their HbA1c screening results. Therefore, this study aimed to evaluate, among those who received no diabetes medications before March 2014, the relationship between HbA1c screening results at the 2012 and 2013 health check-ups and future risk of type 2 diabetes development and worsening within the next 4 years (by March 2018) using health check-up data collected from April 2012 to March 2018 and related health insurance claims.

## Methods

### Study design, population, and data

This retrospective cohort study used the data from the Shizuoka Kokuho Database, an administrative claims database of persons insured by the National Health Insurance (NHI) and Late Elders’ Health Insurance (LEHI), Shizuoka prefecture, Japan. Located approximately at the center of Japan on the Pacific coast, the Shizuoka prefecture has a population of approximately 3.6 million people as of 2020 and is the tenth largest prefecture among 47 prefectures in the country.

There are three main types of public health insurance in Japan, covering almost the entire Japanese population: the Employee’s Health Insurance (EHI), NHI, and LEHI. The EHI and NHI cover those aged ≤74 years, whereas the LEHI covers those aged ≥75 years [11]. The EHI is provided to employed workers (company employees) and their dependents and insured by many insurers (the number of insurers in Japan is more than 1500), mostly dependent on the company’s size. Meanwhile, the NHI is for individuals who are not company employees (hence, not eligible to be members of the EHI), are aged 40–74 years, and are insured by the prefectural and municipal governments (villages, towns, and cities). Those aged > 75 years, including self-employed persons aged > 75 years, are enrolled in the LEHI, which is insured by the prefectures. The Shizuoka Kokuho Database does not contain insurance claims data from the EHI. As of 2018, those insured by NHI and LEHI accounted for 23.1 and 15.0% of the prefecture’s population, respectively [12, 13].

The Shizuoka Kokuho Database also contains data on voluntary health check-ups, which are performed annually as part of the NHI and LEHI systems for those aged > 40 years at designated community centers and medical institutions [11]. A health check-up notification and coupon are mailed to each household on late April based on the city’s family registry. Each person can participate in one health check-up every year, which comprises a physical examination, blood test, and self-reported treatment history with a lifestyle survey. The proportions of NHI- and LEHI-insured individuals who underwent health check-ups in 2018 were 38.7 and 26.5%, respectively [12, 13]. This database does not cover re-examinations or detailed examinations that people voluntarily undergo at medical institutions after the health check-ups.

This study considered both the insurance claims data, which included data on prescribed medications (detailing the year and month of the prescription), and the health check-up data for all insured persons enrolled in the NHI and LEHI in the Shizuoka prefecture between April 2012 and March 2018. Data were tied to individuals using anonymized individual identifiers for research purposes. More details about the database can be found elsewhere [14]. It should be noted that the Japanese fiscal year begins in April and ends in March. Unless indicated otherwise, a calendar year refers to the period from April of that year to March of the following year.

### Participant selection and eligibility

The inclusion criteria were as follows: age > 40 years, availability for follow-up between April 2012 and March 2018 (the study period), with 2012 and 2013 health check-up records including HbA1c levels, and not treated with any diabetes medications before March 2014.

The database included data on the dates when insured persons were enrolled into and withdrew from the NHI and LEHI schemes, which enabled us to identify those who have enrolled from April 2012 to March 2018 (i.e., those who can be followed-up in the Shizuoka Kokuho Database during the period). Insured persons who withdrew during this period included those who transferred their resident cards to another prefecture or those who transferred their insurance to the EHI scheme. Those who died were also excluded from the insurance schemes.

Those who received any diabetes medications before March 2014 were divided into two groups. One group included individuals who self-reported undergoing diabetes treatment or dialysis at the health check-ups in 2012 or 2013, and the other included those prescribed with diabetes medications, including injection drugs (i.e., insulin and glucagon-like peptide-1 [GLP-1] agonists) between 2012 and 2013 based on the insurance claims data [15].

### Main outcomes

The two main outcomes analyzed were diabetes development and worsening, defined as the initiation of diabetes treatment by March 2018 and the use of injection drugs by those patients who initiated diabetes treatment by March 2018, respectively. Treatment initiation was defined as a prescription for a hypoglycemic drug more than once every three months. Whether the treatment was an oral drug or injectable drug was based on the type of drug used in Japan [15]. In the statistical analyses, each outcome was considered as a dependent binary variable.

### Variable of primary interest

A variable of primary interest was the HbA1c screening results for 2012 and 2013. Participants were divided into 4 groups according to HbA1c levels: group A, those whose HbA1c levels were < 6.5% in 2012 and 2013; group B, those whose HbA1c levels > 6.5% in 2012 but < 6.5% in 2013; group C, those whose HbA1c levels were > 6.5% in 2012 and 2013; and group D, those whose HbA1c levels were < 6.5% in 2012 and > 6.5% in 2013. These four groups were determined based on the ease of clinical and policy decision-making and sample size [16, 17]. In the statistical analyses, HbA1c status was considered as an independent categorical covariate with 4 groups.

### Other variables

The other variables collected were data from the 2013 health check-up results, including demographic, clinical, and lifestyle characteristics of the participants. Continuous variables included age, body mass index, systolic blood pressure, diastolic blood pressure, triglycerides (mg/dL), high-density lipoprotein, low-density lipoprotein, fasting blood glucose, glutamate-oxaloacetate transaminase, glutamate-pyruvate transaminase, gamma-glutamyl transpeptidase, hematocrit, hemoglobin, red blood cell, and epidermal growth factor receptor. Categorical variables included sex, urine glucose, urine protein, anti-hypertensive drug use, lipid-lowering drug use, treatment history of cerebrovascular disease, treatment history of cardiovascular disease, daily smoking, and alcohol consumption. In addition, the number of check-ups between April 2014 and March 2018 was considered as an independent categorical covariate, with a range of 0 to 4. This variable was considered as an indicator of the frequency trend of health check-up participation and referred to all check-ups during the period, regardless of treatment initiation.

### Statistical analyses

For descriptive analysis, we compared participant characteristics between treatment initiation group and non-initiation group and among the four HbA1c status groups. Within the treatment initiation group, comparisons were also made among the four HbA1c status groups.

Treatment initiation patterns, including proportions of the treatment initiation group and those who used injection drugs, were compared among the four HbA1c status groups. Patients were further stratified according to age (40–50, 50–60, 60–70, 70–80, and > 80 years old).

The standardized difference score (SDR) was used to test for differences between any unadjusted data and was calculated using the stddiffi command in Stata 17 [18]. Unlike other statistical tests (such as *t*-test and chi-squared test), the SDR approach is not affected by sample size [19] and can be more informative than *p*-values in comparisons between data [20]. A SRD < 0.1 indicates no meaningful difference between the data in relation to the distribution of the variable of interest [21].

Logistic regression models for each of the two binary outcomes (Model 1 for diabetes development and Model 2 for diabetes worsening) were constructed. For both outcomes, variable selection was based on the backward-forward stepwise method with a p-to-remove value > 0.10 and a p-to-entry value < 0.05 . A covariate of primary interest (i.e., HbA1c status) was entered into the models, regardless of significance and as long as stable models were obtained. The reference group for the HbA1c status was set to group A, which had the largest number of participants. For Model 2, data were restricted to the treatment initiation group. A *p*-value less than 0.05 was considered statistically significant.

## Results

**Fig. 1:**
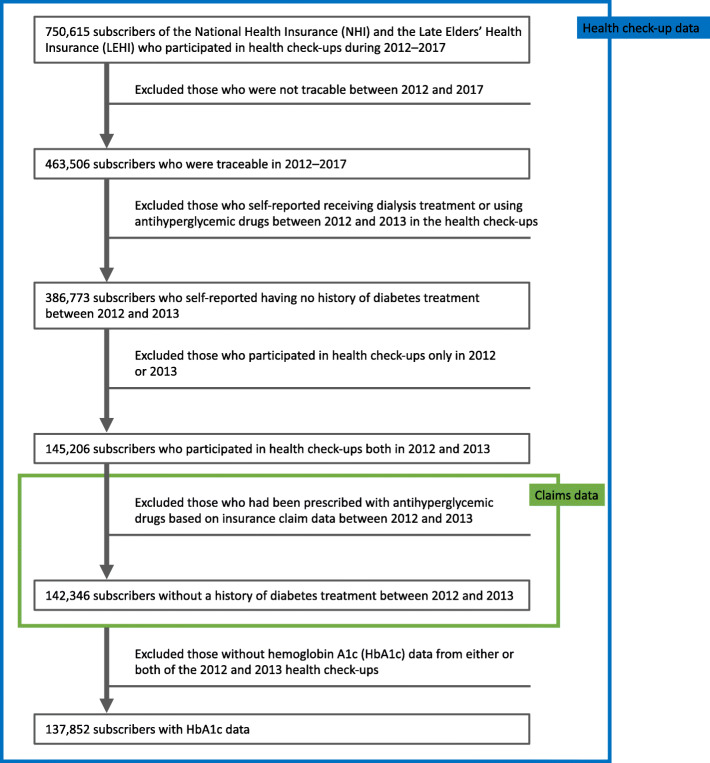
Flowchart of the participant selection process. Note that the Japanese fiscal year begins in April and ends in March. Thus, a calendar year refers to the period from April of that year to March of the following year

Figure 1 shows the flowchart for participant selection. Of 750,615 individuals with health check-up records between April 2012 and March 2018, 463,506 were available for follow-up. As of April 1, 2014, the mean age of participants available for follow-up was 68.05 years (standard deviation [SD] 11.27), whereas that of participants lost to follow-up was 70.52 years (SD 10.24), with an SDR of 0.23. The proportion of female participants available for follow-up was 55.65%, and that lost to follow-up was 57.82%, with an SDR of 0.04. Finally, 137,852 participants met the eligibility criteria and were included in the analysis.

The characteristics of participants in the treatment initiation group (*n* = 3315) and non-initiation group (*n* = 134,537) are presented in Table 1. The mean age of participants in each group was 69.13 years (SD 8.85) and 68.57 (SD 9.89) years, respectively (SDR −0.06). The proportion of female participants was higher in the non-initiation group (women, 57.57%; men, 42.43%), whereas that of male participants was higher in the treatment initiation group (women, 43.89%; men 56.11%), with an SDR of 0.28 (Table 1).

**Table 1:** Demographic characteristics of study participants based on diabetes treatment initiation (*n* = 137,852)

	Treatment initiation group (n = 3315)	Non-initiation group (*n* = 134,537)	Standardized difference score
Demographic characteristics			
Age, years (mean, SD)	69.13 (8.85)	68.57 (9.89)	−0.060
Sex (*n*, %)			0.276
Female	1455 (43.89)	77,449 (57.57)	
Male	1860 (56.11)	57,088 (42.43)	
Clinical characteristics			
BMI (mean, SD)	24.17 (3.69)	22.28 (3.10)	−0.552
SBP, mmHg (mean, SD)	131.98 (15.90)	128.17 (16.26)	−0.237
DBP, mmHg (mean, SD)	76.02 (10.66)	74.47 (10.56)	−0.146
Triglycerides, mg/dL (mean, SD)	141.81 (100.28)	108.76 (65.34)	−0.391
HDL, mg/dL (mean, SD)	56.70 (15.01)	63.52 (16.56)	0.432
LDL, mg/dL (mean, SD)	125.11 (31.03)	124.38 (29.39)	−0.024
FBG, mg/dL (mean, SD)	122.90 (27.69)	94.23 (11.32)	−1.357
GOT, IU/L (mean, SD)	27.07 (14.74)	23.69 (9.06)	−0.276
GPT, IU/L (mean, SD)	27.02 (20.18)	19.35 (11.80)	−0.464
γ-GTP, IU/L (mean, SD)	46.64 (74.68)	30.18 (34.86)	−0.283
HbA1c, % (mean, SD)	6.62 (0.87)	5.57 (0.35)	−1.576
Hematocrit, % (mean, SD)	43.04 (4.06)	41.55 (3.89)	−0.373
Hemoglobin, g/dL (mean, SD)	14.18 (1.48)	13.60 (1.40)	−0.405
RBC, 10^4^/μL (mean, SD)	458.94 (46.97)	441.68 (43.67)	−0.381
Uric acid, mg/dL (mean, SD)	5.41 (1.31)	5.17 (1.34)	−0.184
Serum creatinine, mg/dL (mean, SD)	0.78 (0.28)	0.76 (0.31)	−0.075
eGFR, mL/min (mean, SD)	70.37 (15.64)	69.25 (14.74)	−0.074
Urine glucose (*n*, %)			0.443
Negative	2925 (88.24)	132,761 (98.68)	
Trace	95 (2.87)	665 (0.49)	
1+	109 (3.29)	460 (0.34)	
2+	71 (2.14)	200 (0.15)	
3+	110 (3.32)	164 (0.12)	
Urine protein (*n*, %)			0.205
Negative	2704 (81.57)	118,467 (88.06)	
Trace	333 (10.05)	10,272 (7.64)	
1+	187 (5.64)	4172 (3.10)	
2+	64 (1.93)	1086 (0.81)	
3+	23 (0.69)	250 (0.19)	
Anti-hypertensive drugs (*n*, %)	1750 (52.79)	51,296 (38.13)	0.298
Lipid-lowering drugs (*n*, %)	1090 (32.88)	32,496 (24.15)	0.194
Treatment history (*n*, %)			
Cerebrovascular disease	196 (5.91)	5471 (4.07)	0.085
Cardiovascular disease	277 (8.36)	8384 (6.23)	0.082
Lifestyle characteristics			
Daily smoking (*n*, %)	420 (12.67)	11,875 (8.83)	0.124
Alcohol consumption (*n*, %)			0.047
Daily	719 (21.69)	26,793 (19.91)	
Sometimes	635 (19.16)	25,606 (19.03)	
Never	1788 (53.94)	74,882 (55.66)	

SD, standard deviation; BMI, body mass index; SBP, systolic blood pressure; DBP, diastolic blood pressure; HDL, high-density lipoprotein; LDL, low-density lipoprotein; FBG, fasting blood glucose; GOT, glutamate-oxaloacetate transaminase; GPT, glutamate-pyruvate transaminase; γ-GTP, gamma-glutamyl transpeptidase; HbA1c, hemoglobin A1c; RBC, red blood cell; eGFR, epidermal growth factor receptor

The characteristics of the study participants stratified according to HbA1c status are also presented in Table 2. A total of 133,885 (97.12%), 152 (0.11%), 986 (0.72%), and 2829 (2.05%) participants were included in HbA1c groups A–D, respectively. Participants in groups B–D did not differ from those in the reference group A in terms of mean age (SDR < 0.1 for all groups); however, gender proportions were different among the groups (SDR > 0.1 for all groups). Most clinical and lifestyle data were also different from group A. Similarly, the characteristics of patients stratified according to HbA1c status in the treatment initiation group are also presented in Additional file 1: Appendix Table 1. Of 3315 participants, 1578 (47.60%), 42 (1.27%), 589 (17.77%), and 1106 (33.36%) were included in HbA1c groups A–D, respectively. In comparison with group A, all groups showed differences (SDR > 0.1) in most variables including age.

**Table 2:** Demographic characteristics of study participants stratified according to HbA1c status

	Group A (*n* = 133,885)	Group B (*n* = 152)	SDR*	Group C (*n* = 986)	SDR*	Group D (*n* = 2829)	SDR*
Demographic characteristics							
Age, years (mean, SD)	68.57 (9.90)	67.89 (8.16)	−0.075	68.89 (8.94)	0.034	69.35 (8.26)	0.086
Sex (n, %)			−0.543		− 0.355		− 0.246
Female	77,174 (57.64)	48 (31.58)		396 (40.16)		1286 (45.46)	
Male	56,711 (42.36)	104 (68.42)		590 (59.84)		1543 (54.54)	
Clinical characteristics							
BMI (mean, SD)	22.28 (3.11)	23.56 (2.86)	0.428	23.84 (3.51)	0.472	24.01 (3.43)	0.527
SBP, mmHg (mean, SD)	128.15 (16.25)	129.08 (16.15)	0.057	132.93 (16.77)	0.289	131.73 (16.02)	0.221
DBP, mmHg (mean, SD)	74.46 (10.56)	76.01 (10.36)	0.149	76.94 (10.87)	0.232	75.92 (10.65)	0.138
Triglycerides, mg/dL (mean, SD)	108.61 (65.33)	122.66 (76.28)	0.198	147.54 (118.44)	0.407	140.33 (86.00)	0.415
HDL, mg/dL (mean, SD)	63.54 (16.56)	55.20 (13.46)	−0.553	56.83 (14.53)	−0.431	57.33 (15.30)	− 0.389
LDL, mg/dL (mean, SD)	124.31 (29.39)	120.03 (31.24)	−0.141	130.76 (32.05)	0.210	126.42 (30.10)	0.071
FBG, mg/dL (mean, SD)	93.98 (10.81)	110.63 (12.77)	1.408	143.67 (36.05)	1.867	120.68 (19.245)	1.711
GOT, IU/L (mean, SD)	23.70 (9.11)	22.38 (6.83)	−0.164	25.57 (12.40)	0.172	26.63 (13.27)	0.257
GPT, IU/L (mean, SD)	19.33 (11.82)	21.05 (10.26)	0.155	25.55 (16.30)	0.437	26.82 (19.66)	0.462
γ-GTP, IU/L (mean, SD)	30.20 (35.57)	34.28 (24.55)	0.133	46.28 (54.59)	0.349	42.81 (58.58)	0.260
HbA1c, % (mean, SD)	5.56 (0.32)	6.15 (0.28)	1.970	7.49 (1.11)	2.369	6.75 (0.39)	3.351
Hematocrit, % (mean, SD)	41.55 (3.89)	43.11 (4.26)	0.382	43.44 (4.04)	0.477	42.66 (4.15)	0.275
Hemoglobin, g/dL (mean, SD)	13.60 (1.40)	14.17 (1.49)	0.400	14.36 (1.49)	0.526	14.05 (1.54)	0.309
RBC, 10^4^/μL (mean, SD)	441.55 (43.64)	457.98 (48.12)	0.358	464.02 (47.01)	0.496	459.46 (45.63)	0.401
Uric acid, mg/dL (mean, SD)	5.17 (1.34)	5.52 (1.23)	0.275	5.22 (1.29)	0.042	5.45 (1.30)	0.217
Serum creatinine, mg/dL (mean, SD)	0.76 (0.31)	0.83 (0.47)	0.173	0.76 (0.19)	0.000	0.77 (0.20)	0.053
eGFR, mL/min (mean, SD)	69.23 (14.74)	70.57 (14.99)	0.090	72.84 (16.13)	0.234	70.28 (15.09)	0.070
Urine glucose (n, %)			−0.344		−0.791		−0.395
Negative	132,268 (98.79)	139 (91.45)		727 (73.73)		2552 (90.21)	
Trace	617 (0.46)	5 (3.29)		63 (6.39)		75 (2.65)	
1+	410 (0.31)	2 (1.32)		70 (7.10)		87 (3.08)	
2+	168 (0.13)	2 (1.32)		53 (5.38)		48 (1.70)	
3+	136 (0.10)	3 (1.97)		72 (7.30)		63 (2.23)	
Urine protein (n, %)			−0.205		−0.285		−0.154
Negative	117,900 (88.06)	125 (82.24)		769 (77.99)		2377 (84.02)	
Trace	10,228 (7.64)	14 (9.21)		122 (12.37)		241 (8.52)	
1+	4146 (3.10)	8 (5.26)		66 (6.69)		139 (4.91)	
2+	1073 (0.80)	4 (2.63)		20 (2.03)		53 (1.87)	
3+	249 (0.19)	0 (0.00)		8 (0.81)		16 (0.57)	
Anti-hypertensive drugs (n, %)	51,127 (38.19)	69 (45.39)	−0.147	415 (42.09)	−0.080	1435 (50.72)	−0.254
Lipid-lowering drugs (n, %)	32,381 (24.19)	34 (22.37)	−0.043	241 (24.44)	−0.006	930 (32.87)	−0.193
Treatment history (n, %)							
Cerebrovascular disease	5470 (4.09)	7 (4.61)	−0.025	45 (4.56)	−0.023	145 (5.13)	−0.050
Cardiovascular disease	8348 (6.24)	7 (4.61)	−0.072	65 (6.59)	−0.014	241 (8.52)	−0.088
Lifestyle characteristics							
Daily smoking (n, %)	11,842 (8.84)	23 (15.13)	−0.194	141 (14.3)	−0.171	289 (10.22)	−0.047
Alcohol consumption (n, %)			−0.149		−0.130		− 0.043
Daily	26,634 (19.89)	38 (25.00)		249 (25.25)		591 (20.89)	
Sometimes	25,458 (19.01)	28 (18.42)		188 (19.07)		567 (20.04)	
Never	74,569 (55.7)	74 (48.68)		507 (51.42)		1520 (53.73)	

* SDR values are calculated with group A as reference. SDR, standardized difference score; SD, standard deviation, BMI, body mass index; SBP, systolic blood pressure; DBP, diastolic blood pressure; HDL, high-density lipoprotein; LDL, low-density lipoprotein; FBG, fasting blood glucose; GOT, glutamate-oxaloacetate transaminase; GPT, glutamate-pyruvate transaminase; γ-GTP, gamma-glutamyl transpeptidase; HbA1c, hemoglobin A1c; RBC, red blood cell; eGFR, epidermal growth factor receptor

The treatment initiation patterns are shown in Table 3. In HbA1c groups A–D, the proportions of participants who initiated diabetes treatment were 1.18, 27.63, 59.74, and 39.10%, respectively (SDR > 0.1 for all comparisons versus group A). Among the treatment initiation group, the proportions of participants using injection drugs in HbA1c groups A–D were 2.85, 7.14, 3.23, and 0.99%, respectively; the SDR values of these proportions between groups B and D versus group A were > 0.1. Similar treatment patterns were observed in the age subgroups (Additional file 1: Appendix Table 2).

**Table 3:** Treatment initiation patterns for all ages stratified according to HbA1c status

All ages	Group A	Group B	Group C	Group D	Total
Total (n, % in row)	133,885 (97.12)	152 (0.11)	986 (0.72)	2829 (2.05)	137,852 (100.00)
Not initiated (n, %)	132,307 (98.82)	110 (72.37)	397 (40.26)	1723 (60.90)	134,537 (97.60)
Initiated (n, %)	1578 (1.18)	42 (27.63)	589 (59.74)	1106 (39.10)	3315 (2.40)
Oral drug only (n, %)	1533 (97.15)	39 (92.86)	570 (96.77)	1095 (99.01)	3237 (97.65)
Injection drug (n, %)	45 (2.85)	3 (7.14)	19 (3.23)*	11 (0.99)	78 (2.35)

In comparison with group A, groups B–D showed differences (SDR > 0.1) in both the proportions of the treatment initiated group as well as the proportions of those who ended up using injection drugs among the treatment initiated group, except for *. HbA1c, hemoglobin A1c. Participants were divided into 4 groups according to HbA1c levels: group A, those whose HbA1c levels were < 6.5% in 2012 and 2013; group B, those whose HbA1c levels > 6.5% in 2012 but < 6.5% in 2013; group C, those whose HbA1c levels were > 6.5% in 2012 and 2013; and group D, those whose HbA1c levels were < 6.5% in 2012 and > 6.5% in 2013

The results of the logistic regression analyses are shown in Table 4. In Model 1, after adjusting for covariates, the diabetes treatment initiation was found to be statistically significantly associated with the HbA1c screening results. Compared with HbA1c ggoup A, the other HbA1c groups had higher odds of treatment initiation, with the following odds ratios (ORs): 22.64 (95% confidence interval [CI] 14.66–34.99), 90.83 (95% CI 76.33–108.08), and 36.95 (95% CI 33.10–41.26) for groups B–D, respectively.

**Table 4:** Adjusted odds ratios*

	Model 1		Model 2	
Variables	Odds ratio (95% CI)	p-value	Odds ratio (95% CI)	p-value
HbA1c status				
Group A	Ref.		Ref.	
Group B	22.64 (14.66–34.99)	< 0.001	3.02 (0.84–10.97)	0.09
Group C	90.83 (76.33–108.08)	< 0.001	0.54 (0.25–1.18)	0.12
Group D	36.95 (33.10–41.26)	< 0.001	0.28 (0.12–0.61)	< 0.01
Number of health check-ups between April 2014 and March 2018				
0	Ref.		Ref.	
1	0.84 (0.66–1.08)	0.19	0.52 (0.20–1.38)	0.19
2	0.99 (0.79–1.25)	0.94	0.45 (0.18–1.14)	0.09
3	1.04 (0.84–1.29)	0.73	0.24 (0.09–0.61)	< 0.01
4	0.88 (0.72–1.07)	0.20	0.19 (0.09–0.42)	< 0.001
Age, years	1.01 (1.00–1.01)	0.08	0.98 (0.95–1.01)	0.17
Sex				
Female	1.05 (0.92–1.20)	0.48	1.13 (0.63–2.05)	0.68
Male	Ref.		Ref.	
BMI	1.08 (1.07–1.10)	< 0.001	–	
SBP, mmHg	1.01 (1.00–1.01)	< 0.001	–	
DBP, mmHg	0.99 (0.98–0.99)	< 0.001	–	
Triglyceride, mg/dL	1.00 (1.00–1.00)	< 0.01		
HDL, mg/dL	0.99 (0.99–0.99)	< 0.001	1.02 (1.00–1.03)	0.06
GOT, IU/L	0.99 (0.99–1.00)	0.06	–	
GPT, IU/L	1.01 (1.01–1.02)	< 0.001	–	
γ-GTP, IU/L	1.00 (1.00–1.00)	< 0.001	–	
Hemoglobin, g/dL	1.10 (1.05–1.14)	< 0.001	–	
Uric acid, mg/dL	–		–	
Serum creatinine, mg/dL	1.31 (0.99–1.71)	0.06	–	
eGFR, mL/min	1.01 (1.00–1.01)	< 0.001		
Urine glucose				
Negative	Ref.		Ref.	
Trace	1.19 (0.84–1.69)	0.33	3.18 (1.01–10.00)	< 0.05
1+	1.69 (1.20–2.37)	< 0.01	1.27 (0.29–5.64)	0.75
2+	2.47 (1.55–3.92)	< 0.001	4.72 (1.45–15.36)	< 0.05
3+	2.39 (1.58–3.63)	< 0.001	2.65 (0.83–8.44)	0.10
Urine protein (n, %)				
Negative	Ref.		Ref.	
Trace	1.14 (0.97–1.33)	0.10	1.39 (0.60–3.22)	0.44
1+	1.27 (1.02–1.57)	< 0.05	2.24 (0.92–5.47)	0.08
2+	1.21 (0.82–1.80)	0.34	1.89 (0.42–8.49)	0.41
3+	1.59 (0.85–2.98)	0.15	–	
Anti-hypertensive drugs	1.32 (1.19–1.47)	< 0.001	–	
Lipid-lowering drugs	1.37 (1.24–1.52)	< 0.001	0.37 (0.17–0.78)	< 0.01
Treatment history				
Cerebrovascular disease	1.19 (0.97–1.46)	0.09	–	
Daily smoking	1.14 (0.98–1.33)	0.08	–	

* Diabetes treatment initiation versus non-initiation in all participants (Model 1) and injection drug use versus oral drug use in treatment initiation group (Model 2). CI, confidence interval; HbA1c, hemoglobin A1c; BMI, body mass index; SBP, systolic blood pressure; DBP, diastolic blood pressure; HDL, high-density lipoprotein; GOT, glutamate-oxaloacetate transaminase; GPT, glutamate-pyruvate transaminase; γ-GTP, gamma-glutamyl transpeptidase; eGFR, epidermal growth factor receptor. Participants were divided into 4 groups according to HbA1c levels: group A, those whose HbA1c levels were < 6.5% in 2012 and 2013; group B, those whose HbA1c levels > 6.5% in 2012 but < 6.5% in 2013; group C, those whose HbA1c levels were > 6.5% in 2012 and 2013; and group D, those whose HbA1c levels were < 6.5% in 2012 and > 6.5% in 2013

In Model 2, after adjusting for covariates, there was no statistically significant difference in the odds of the use of injection drugs between group A and groups B and C (Table 4). Lower odds of the use of injection drugs was observed in group D relative to group A (OR 0.28, 95% CI 0.12–0.61). From April 2014 to March 2018, those who underwent at least three health check-ups had lower odds of the use of injection drugs than those who never received one, with an OR of 0.24 (95% CI 0.09–0.61) for participants who underwent three health check-ups and an OR of 0.19 (95% CI 0.09–0.42) for participants who underwent four health check-ups.

## Discussion

We found that among those who self-reported no history of diabetes treatment at the 2012 and 2013 health check-ups and had no history of diabetes prescriptions in these years, HbA1c screening results were statistically significantly associated with the initiation of diabetes treatment within 4 years (Model 1 in Table 4), after adjusting for covariates. This result is in line with scientific evidence that HbA1c alone is not necessarily sensitive enough to identify prediabetics but correlated well with the future risk of developing diabetes [22]. Compared to the HbA1c group A, group B was more likely to start treatment for diabetes within 4 years. This finding echoes the importance that even if the HbA1c level is normal at the time of measurement, the previous years’ screening results should be taken into account, and the possibility that people like Group B are prediabetic should be carefully considered. Physicians and patients need to pay sufficient attention to lifestyle habits and other factors related to diabetes, including diet and inadequate lifestyle modification that could result in treatment initiation [23].

Moreover, among people who initiated diabetes treatment by March 2018, the odds of the use of injection drugs in group A were not statistically significantly different from those in group C, and were significantly higher than in those in group D (Model 2 in Table 4) after adjusting for covariates. This finding also suggests that although the HbA1c level was normal at the time of measurement, it does not necessarily guarantee a low risk of diabetes worsening. Thus, insufficient attention to lifestyle habits could easily lead to a deterioration in glycemic control and result in the initiation of injection drug treatment [24, 25].

Our study also found that participants who underwent health check-ups annually were less likely to initiate injection drug treatment (Model 2 in Table 4). The number of health check-ups was used as an indicator of the frequency of health check-up participation. This finding may not necessarily demonstrate the causal relationship between health check-up participation and diabetes worsening; instead, it may be attributed to the fact that participants who are more concerned about their health are more likely to undergo health check-ups more frequently [26]. Alternatively, it is possible that participants who started using injection drugs no longer felt the need for health check-ups. However, previous studies have suggested that health check-ups might be useful in screening for lifestyle-related diseases up to a certain extent [27–29], and that they will continue to play a role in the early detection of the onset and the worsening of diabetes.

To the best of our knowledge, this is the first study to have followed up Japanese adults to assess their risk of diabetes development or worsening based on HbA1c screening results. However, our study had some limitations. We defined diabetes development and worsening as initiation of diabetes treatment by March 2018 and the use of injection drugs in those who initiated diabetes treatment by March 2018, respectively, because these were the only definitions possible for our data. Therefore, our outcomes were not exactly the onset or diagnosis of diabetes, or worse in the stricter sense, uncontrolled diabetes. For example, our data lacked information on the implementation of lifestyle guidance such as exercise and diet, HbA1c levels at the time of hospital visits, or data that would accurately suggest the onset of complications. Regarding the possible selection bias in study participants, participating in annual health check-ups is voluntary; therefore, individuals more concerned about their health were more likely to be included in the study. Furthermore, the health check-up data were limited to those aged > 40 years, and data of insured individuals enrolled in the EHI scheme were not considered. Only participants in the Shizuoka prefecture were also included in the study, which was not necessarily representative of the entire Japanese population. Therefore, our findings may not be generalizable to a wider population.

In addition, we considered only those that could be tracked between April 2012 and March 2018 in the Shizuoka Kokuho Database, which may create survival bias, thus suggesting the possible underestimates of diabetes risks in our data. In fact, the mean age of the participants available for follow-up was lower than that of participants lost to follow-up (SDR > 0.1). In addition to death, the main causes of lost to follow-up included transfer of resident registration to another prefecture and shift to the EHI system. These cases cannot be confirmed for each individual in the database.

Furthermore, although GLP-1 agonists were classified as injection drugs in this study, GLP-1 agonists are usually used as first-line agents in Japan. Insulin, also classified as an injection drug, is mainly used for patients with severe diabetes. Thus, although GLP-1 agonists are injection drugs, they may not be appropriate indicators of the severity of diabetes. GLP-1 agonists were intentionally classified as an oral drug for sensitivity analysis; however, the results remain unchanged. One possible reason for this may be that during the study period, the introduction of GLP-1 agonists was not yet widespread, and only a few patients in the Shizuoka prefecture received GLP-1 agonist prescription. Finally, patients with type 1 and 2 diabetes were not differentiated in our study. However, most participants aged ≥40 years who were diagnosed with diabetes were likely to have type 2 diabetes because of its higher prevalence in the general population (approximately 95%) [30, 31]; hence, given that all participants in this study were aged > 40 years, it can be assumed that most participants who initiated treatment had type 2 diabetes.

## Conclusions

Our study suggests that although HbA1c levels measured during health check-ups were correlated with the risk of diabetes development and worsening, HbA1c measurements based in a single year may not necessarily provide sufficient information to predict the risk of diabetes development and worsening. Guidance on preventive health behaviors and lifestyle measures to lower the risk of diabetes development and worsening should still be imparted to individuals with normal HbA1c levels during health check-ups.

## Supplementary Information

**Additional file 1: Appendix Table 1.** Demographic characteristics of study participants who started diabetes treatment by March 2018 stratified according to HbA1c status**. Appendix Table 2.** Treatment initiation patterns for age subgroups stratified according to HbA1c status.

## Data Availability

The data that support the findings of this study are available from the Shizuoka General Hospital, but restrictions apply to the availability of these data, which were used under license for the current study, and so are not publicly available. Data are, however, available from the corresponding author upon reasonable request and with permission of the Shizuoka General Hospital.

## Abbreviations

GBD: Global Burden of Disease
BMI: body mass index
HbA1c: hemoglobin A1c
NHI: National Health Insurance
LEHI: Late Elders’ Health Insurance
EHI: Employee’s Health Insurance
SDR: Standardized difference score
SD: Standard deviation
GLP-1: glucagon-like peptide-1
