# Supplementary material for: Analysis of the relationship between the HbA1c screening results and the development and worsening of diabetes among adults aged over 40 years: a 4-year follow-up study of 140,000 people in Japan – the Shizuoka study
Source: BMC Public Health. 2021 Oct 18;21:1880. doi: 10.1186/s12889-021-11933-z (PMC8524880; doi:10.1186/s12889-021-11933-z)
Supplement: Supplementary file 1 — Additional file 1: Appendix Table 1. Demographic characteristics of study participants who started diabetes treatment by March 2018 stratified according to HbA1c status. Appendix Table 2. Treatment initiation patterns for age subgroups stratified according to HbA1c status. [file 12889_2021_11933_MOESM1_ESM.docx]

**Appendix Table 1: Demographic characteristics of study participants who started diabetes treatment by March 2018 stratified according to HbA1c status**

|  | Group A  (n=1,578) | Group B  (n=42) |  | Group C  (n=589) |  | Group D  (n=1,106) |  |
| --- | --- | --- | --- | --- | --- | --- | --- |
|  |  |  | SDR* |  | SDR* |  | SDR* |
| Demographic characteristics |  |  |  |  |  |  |  |
| Age, years (mean, SD) | 69.49 (9.10) | 67.67 (7.31) | -0.221 | 68.36 (8.94) | -0.125 | 69.08 (8.47) | -0.046 |
| Sex (n, %) |  |  | -0.363 |  | -0.082 |  | -0.060 |
| Female | 723 (45.82) | 12 (28.57) |  | 246 (41.77) |  | 474 (42.86) |  |
| Male | 855 (54.18) | 30 (71.43) |  | 343 (58.23) |  | 632 (57.14) |  |
| Clinical characteristics |  |  |  |  |  |  |  |
| BMI (mean, SD) | 24.06 (3.78) | 23.67 (3.37) | -0.108 | 23.87 (3.64) | -0.052 | 24.50 (3.59) | 0.121 |
| SBP, mmHg (mean, SD) | 131.40 (15.83) | 128.20 (15.33) | -0.210 | 132.90 (17.17) | 0.087 | 132.40 (15.29) | 0.065 |
| DBP, mmHg (mean, SD) | 75.35 (10.57) | 75.88 (7.63) | 0.057 | 76.61 (10.98) | 0.117 | 76.65 (10.66) | 0.122 |
| Triglycerides, mg/dL (mean, SD) | 136.60 (106.21) | 114.00 (57.20) | -0.265 | 147.60 (112.13) | 0.100 | 147.20 (84.44) | 0.110 |
| HDL, mg/dL (mean, SD) | 57.21 (15.65) | 54.50 (17.09) | -0.165 | 56.58 (14.25) | -0.042 | 56.12 (14.39) | -0.072 |
| LDL, mg/dL (mean, SD) | 120.80 (30.71) | 116.20 (32.06) | -0.145 | 131.50 (31.47) | 0.346 | 128.20 (30.27) | 0.244 |
| FBG, mg/dL (mean, SD) | 109.49 (14.87) | 116.41 (12.59) | 0.502 | 150.70 (39.22) | 1.390 | 126.31 (21.48) | 0.910 |
| GOT, IU/L (mean, SD) | 26.83 (14.66) | 23.21 (7.32) | -0.312 | 25.56 (13.15) | -0.091 | 28.35 (15.72) | 0.100 |
| GPT, IU/L (mean, SD) | 25.60 (19.33) | 24.05 (12.95) | -0.094 | 25.97 (17.18) | 0.020 | 29.72 (22.68) | 0.196 |
| γ-GTP, IU/L (mean, SD) | 44.32 (74.97) | 34.60 (19.19) | -0.178 | 47.19 (61.57) | 0.042 | 50.12 (81.55) | 0.074 |
| HbA1c, % (mean, SD) | 6.07 (0.29) | 6.20 (0.36) | 0.419 | 7.68 (1.25) | 1.772 | 6.85 (0.44) | 2.119 |
| Hematocrit, % (mean, SD) | 42.74 (4.13) | 42.77 (4.54) | 0.008 | 43.49 (3.95) | 0.188 | 43.24 (3.96) | 0.123 |
| Hemoglobin, g/dL (mean, SD) | 14.03 (1.48) | 14.08 (1.54) | 0.029 | 14.38 (1.44) | 0.238 | 14.29 (1.48) | 0.173 |
| RBC, 10^4^/μL (mean, SD) | 453.70 (46.99) | 453.10 (52.85) | -0.012 | 465.00 (48.59) | 0.236 | 463.50 (45.04) | 0.212 |
| Uric acid, mg/dL (mean, SD) | 5.48 (1.31) | 5.47 (1.28) | -0.006 | 5.16 (1.28) | -0.248 | 5.46 (1.33) | -0.016 |
| Serum creatinine, mg/dL (mean, SD) | 0.80 (0.35) | 0.80 (0.17) | -0.001 | 0.75 (0.20) | -0.169 | 0.78 (0.20) | -0.065 |
| eGFR, mL/min (mean, SD) | 68.95 (15.18) | 71.32 (14.02) | 0.162 | 73.94 (16.36) | 0.316 | 70.48 (15.69) | 0.099 |
| Urine glucose (n, %) |  |  | -0.309 |  | -0.677 |  | -0.264 |
| Negative | 1,498 (94.93) | 37 (88.10) |  | 419 (71.14) |  | 971 (87.79) |  |
| Trace | 23 (1.46) | 2 (4.76) |  | 36 (6.11) |  | 34 (3.07) |  |
| 1+ | 24 (1.52) | 2 (4.76) |  | 42 (7.13) |  | 41 (3.71) |  |
| 2+ | 10 (0.63) | 0 (0.00) |  | 37 (6.28) |  | 24 (2.17) |  |
| 3+ | 20 (1.27) | 1 (2.38) |  | 54 (9.17) |  | 35 (3.16) |  |
| Urine protein (n, %) |  |  | -0.133 |  | -0.180 |  | -0.069 |
| Negative | 1,313 (83.21) | 34 (80.95) |  | 450 (76.40) |  | 907 (82.01) |  |
| Trace | 150 (9.51) | 5 (11.90) |  | 75 (12.73) |  | 103 (9.31) |  |
| 1+ | 79 (5.01) | 2 (4.76) |  | 43 (7.30) |  | 63 (5.70) |  |
| 2+ | 26 (1.65) | 1 (2.38) |  | 13 (2.21) |  | 24 (2.17) |  |
| 3+ | 7 (0.44) | 0 (0.00) |  | 7 (1.19) |  | 9 (0.81) |  |
| Anti-hypertensive drugs (n, %) | 886 (56.15) | 18 (42.86) | -0.268 | 248 (42.11) | -0.284 | 598 (54.07) | -0.042 |
| Lipid-lowering drugs (n, %) | 572 (36.25) | 6 (14.29) | -0.522 | 146 (24.79) | -0.251 | 366 (33.09) | -0.066 |
| Treatment history (n, %) |  |  |  |  |  |  |  |
| Cerebrovascular disease | 103 (6.53) | 2 (4.76) | -0.077 | 20 (3.40) | -0.145 | 71 (6.42) | -0.004 |
| Cardiovascular disease | 147 (9.32) | 2 (4.76) | -0.179 | 37 (6.28) | -0.114 | 91 (8.23) | -0.038 |
| Lifestyle characteristics |  |  |  |  |  |  |  |
| Daily smoking (n, %) | 198 (12.55) | 3 (7.14) | -0.182 | 84 (14.26) | -0.050 | 135 (12.21) | -0.010 |
| Alcohol consumption (n, %) |  |  | -0.089 |  | -0.064 |  | -0.047 |
| Daily | 328 (20.79) | 10 (23.81) |  | 138 (23.43) |  | 243 (21.97) |  |
| Sometimes | 295 (18.69) | 7 (16.67) |  | 112 (19.02) |  | 221 (19.98) |  |
| Never | 867 (54.94) | 22 (52.38) |  | 312 (52.97) |  | 587 (53.07) |  |

* SDR values are calculated with group A as reference. SDR, standardized difference score; SD, standard deviation, BMI, body mass index; SBP, systolic blood pressure; DBP, diastolic blood pressure; HDL, high-density lipoprotein; LDL, low-density lipoprotein; FBG, fasting blood glucose; GOT, glutamate-oxaloacetate transaminase; GPT, glutamate-pyruvate transaminase; γ-GTP, gamma-glutamyl transpeptidase; HbA1c, hemoglobin A1c; RBC, red blood cell; eGFR, epidermal growth factor receptor.

**Appendix Table 2: Treatment initiation patterns for age subgroups stratified according to HbA1c status**

| 40–50 years old | Group A | Group B | Group C | Group D | Total |
| --- | --- | --- | --- | --- | --- |
| Total (n, % in row) | 5,759 (98.68) | 4 (0.07) | 26 (0.45) | 47 (0.81) | 5,836 (100.00) |
| Not initiated (n, %) | 5,717 (99.27) | 3 (75.00) | 6 (23.08) | 24 (51.06) | 5,750 (98.53) |
| Initiated (n, %) | 42 (0.73) | 1 (25.00) | 20 (76.92) | 23 (48.94) | 86 (1.47) |
| Oral drug only (n, %) | 41 (97.62) | 0 (0.00) | 17 (85.00) | 23 (100.00) | 81 (94.19) |
| Injection drug (n, %) | 1 (2.38) | 1 (100.00) | 3 (15.00) | 0 (0.00) | 5 (5.81) |
|  |  |  |  |  |  |
| 50–60 years old | Group A | Group B | Group C | Group D | Total |
| Total (n, % in row) | 12,002 (97.96) | 13 (0.11) | 72 (0.59) | 165 (1.35) | 12,252 (100.00) |
| Not initiated (n, %) | 118,79 (98.98) | 9 (69.23) | 24 (33.33) | 96 (58.18) | 12,008 (98.01) |
| Initiated (n, %) | 123 (1.02) | 4 (30.77) | 48 (66.67) | 69 (41.82) | 244 (1.99) |
| Oral drug only (n, %) | 119 (96.75) | 4 (100.00) | 44 (91.67) | 69 (100.00) | 236 (96.72) |
| Injection drug (n, %) | 4 (3.25) | 0 (0.00) | 4 (8.33) | 0 (0.00) | 8 (3.28) |
|  |  |  |  |  |  |
| 60–70 years old | Group A | Group B | Group C | Group D | Total |
| Total (n, % in row) | 65,307 (96.88) | 93 (0.14) | 509 (0.76) | 1,504 (2.23) | 67,413 (100.00) |
| Not initiated (n, %) | 64,560 (98.86) | 68 (73.12) | 192 (37.72) | 913 (60.70) | 65,733 (97.51) |
| Initiated (n, %) | 747 (1.14) | 25 (26.88) | 317 (62.28) | 591 (39.30) | 1,680 (2.49) |
| Oral drug only (n, %) | 724 (96.92) | 23 (92.00) | 313 (98.74) | 584 (98.82) | 1,644 (97.86) |
| Injection drug (n, %) | 23 (3.08) | 2 (8.00) | 4 (1.26) | 7 (1.18) | 36 (2.14) |
|  |  |  |  |  |  |
| 70–80 years old | Group A | Group B | Group C | Group D | Total |
| Total (n, % in row) | 28,827 (96.71) | 25 (0.08) | 239 (0.80) | 717 (2.41) | 29,808 (100.00) |
| Not initiated (n, %) | 28,417 (98.58) | 17 (68.00) | 115 (48.12) | 444 (61.92) | 28,993 (97.27) |
| Initiated (n, %) | 410 (1.42) | 8 (32.00) | 124 (51.88) | 273 (38.08) | 815 (2.73) |
| Oral drug only (n, %) | 399 (97.32) | 8 (100.00) | 121 (97.58)* | 272 (99.63) | 800 (98.16) |
| Injection drug (n, %) | 11 (2.68) | 0 (0.00) | 3 (2.42) | 1 (0.37) | 15 (1.84) |
|  |  |  |  |  |  |
| >80 years old | Group A | Group B | Group C | Group D | Total |
| Total (n, % in row) | 21,990 (97.55) | 17 (0.08) | 140 (0.62) | 396 (1.76) | 22,543 (100.00) |
| Not initiated (n, %) | 21,734 (98.84) | 13 (76.47) | 60 (42.86) | 246 (62.12) | 22,053 (97.83) |
| Initiated (n, %) | 256 (1.16) | 4 (23.53) | 80 (57.14) | 150 (37.88) | 490 (2.17) |
| Oral drug only (n, %) | 250 (97.66) | 4 (100.00) | 75 (93.75) | 147 (98.00)* | 476 (97.14) |
| Injection drug (n, %) | 6 (2.34) | 0 (0.00) | 5 (6.25) | 3 (2.00) | 14 (2.86) |

In comparison with group A, groups B–D showed differences (SDR >0.1) in both the proportions of the treatment initiated group as well as the proportions of those who ended up using injection drugs among the treatment initiated group, except for *. HbA1c, hemoglobin A1c. Participants were divided into 4 groups according to HbA1c levels: group A, those whose HbA1c levels were <6.5% in 2012 and 2013; group B, those whose HbA1c levels >6.5% in 2012 but <6.5% in 2013; group C, those whose HbA1c levels were >6.5% in 2012 and 2013; and group D, those whose HbA1c levels were <6.5% in 2012 and >6.5% in 2013.
